# Teachers’ Attitudes towards and Comfort about Teaching School-Based Sexuality Education in Urban and Rural Tanzania

**DOI:** 10.5539/gjhs.v4n4p149

**Published:** 2012-06-20

**Authors:** Kitila Alexander Mkumbo

**Affiliations:** 1 University of Dar es Salaam, United Republic of Tanzania

**Keywords:** Tanzania, sexuality education, attitudes, teachers

## Abstract

Teachers’ attitudes towards sexuality education are among the important predictors of their willingness to teach sexuality education programmes in schools. While there is a plethora of studies on teachers’ attitudes towards sexuality in developed countries, there is a paucity of such studies in sub-Saharan Africa in general and Tanzania in particular. This study examined teachers’ attitudes towards and comfort in teaching sexuality education in rural and urban Tanzania. The results show that an overwhelming majority of teachers in both rural and urban districts supported the teaching of sexuality education in schools, and the inclusion of a wide range of sexuality education topics in the curriculum. Nevertheless, though teachers expressed commitment to teaching sexuality education in schools, they expressed difficult and discomfort in teaching most of the key sexuality education topics. This implies that declaration of positive attitudes towards teaching sexuality education alone is not enough; there is a need for facilitating teachers with knowledge, skills and confidence to teach various sexuality education topics.

## 1. Introduction

The effectiveness of school-based sexuality education depends on, among other factors, the effectiveness of teachers who implement it (Cohen, Sears, Byers, & Weaver, 2004). Furthermore, it has been argued that the extent to which teachers implement the school-based sexuality education curriculum is largely dependent upon and influenced by their attitudes towards it (Paulussen, Kok, & Schaalma, 1994). Indeed, one of the central characteristics of an effective sexuality education programme is the level at which teachers are willing and show positive attitudes towards teaching it (Kirby et al., 2005). It is in this context that several authors have recommended that teachers’ attitudes and confidence about teaching sexuality education be assessed prior to engaging them in the delivery of sexuality education programmes (Paulussen et al., 1994; Oshi, Nakalema & Oshi, 2004; Mathews, Boon, Flisher, & Schaalma, 2006).

In many countries, teachers are perceived by young people as the most credible and trustworthy source of information about sexuality and stand high in the list of young people’s preferences of sexuality education deliverers (Milton, 2003). Thus it is important to take into account teachers’ views and attitudes in the sexuality education programme development and implementation.

Studies have shown that, although teachers in different countries generally support the teaching of sexuality education in schools, they encounter several obstacles. Firstly, teachers often express difficulties in teaching some of the topics related to sexuality education, including condom use, masturbation, sexual orientation, abortion and contraception (Donovan, 1998; Milton, 2003; Munodawafa, 1991). Hudson (1999) has observed that most teachers have no problems delivering sexuality education that consists of biological facts, but they find it difficult facilitating topics related to psychological aspects of sexuality education, including discussion about young people’s opinions, hopes, expectation and fears concerning relationships. An evaluation of the teacher training for AIDS prevention programme in South Africa revealed that teachers, like parents, expressed open resistance to the teaching of condom use on the grounds that condom promotion would encourage promiscuity (Ahmed et al., 2006).

Secondly, there are concerns that teachers find it difficult to move beyond didactic methods in their teaching of sexuality education; they particularly express difficulties in handling participatory facilitation skills that have been found to be effective in the teaching of sexuality education, such as discussions, group work and role - plays (Buston, Wight, Hart, & Scott, 2002).

Thirdly, teachers have expressed concerns that they do not receive enough support from their colleagues and parents when it comes to teaching sexuality education in schools. A study of teachers’ views about teaching sexuality education in 17 secondary schools in one city in England found that teachers reported facing criticism and resentment from other staff and parents about having to teach sexuality education (Alldred, David, & Smith, 2003). This means that, without the support of fellow teachers and the community in general, most teachers may find it difficult teaching sexuality education to children.

It is clear therefore that, as much as teachers may support the teaching of sexuality education in schools, they may lack the knowledge, skills and confidence to handle sexuality education sessions in a classroom situation. Additionally, it has been observed that the training teachers receive in conventional teacher training colleges may be insufficient in so far as teaching of sexuality education is concerned (Vavrus, 2006; Csincsak, Bourdeaudhuij, & Van Oost, 1994). There is, therefore, a need to provide special training tailored to sexuality education if teachers are to handle sexuality education topics effectively and competently in the classroom. Furthermore, there is a need for a clear policy that supports the teaching of comprehensive sexuality education in schools as well as social support from colleagues and community members. The lack of a clear policy on sexuality education and social support from colleagues and teachers, alongside overcrowded classrooms, have been observed as critical barriers to teachers’ successful implementation of school-based sexuality education (Csincsak et al., 1994).

Though there has been a plethora of studies that have examined the attitudes of teachers towards teaching sexuality education in schools in developed countries, there has been a paucity of such studies in sub-Saharan Africa. This study examined teachers’ attitudes towards and comfort about teaching sexuality education in schools in rural and urban Tanzania. Specific objectives of the study were to: assess the attitudes of teachers towards teaching sexuality education; investigate their comfort and confidence in teaching sexuality education; examine the variation in attitudes towards sexuality education between teachers in urban and rural based schools; and assess the effect of demographic factors in influencing teachers’ attitudes towards and comfort in teaching sexuality education.

## 2. Method

### 2.1 Participants

A randomly selected sample of 102 teachers drawn from 12 schools in the urban district (Kinondoni) and 96 teachers in eight schools in the rural district (Sengerema) were requested to complete a survey, with an overall response rate for both districts being 81.4 percent. Table 1 summarises the demographic characteristics of responding teachers.

**Table 1:** Demographic characteristics of responding teachers by district

Demographic variables	% of Respondents	

	Urban District (Kinondoni) N= 102	Rural District (Sengerema) N= 96
Age		
25-35	57.9	30.0
36-45	21.1	23.3
46-55	21.1	36.7
Over 55 years	-	10.0
Sex		
Male	36.1	44.8
Female	63.9	55.2
Religion		
Catholics	39.5	50.0
Protestant	42.1	40.0
Islam	18.4	3.3
Other	-	3.3
None	-	3.3
How many times do you attend religious services		
Everyday	23.7	16.7
At least once a week	73.7	76.7
At least once a month	2.6	3.3
At least once a year	-	3.3
Never attend	-	-
How important is religion in your life		
Very important	94.7	89.3
Important	5.3	10.7
Somehow important	-	-
Not important	-	-
Not important at all	-	-
Teaching qualification		
Certificate in education (Grade A)	92.1	73.3
Certificate in education (Grade B)	-	20.0
Diploma in education	2.6	3.3
University degree	-	3.3
No formal teaching qualification	5.3	-
Teaching experience		
Less than 5 years	42.1	23.3
5-10 years	10.5	6.7
10-15 years	23.7	16.7
More than 15 years	23.7	53.3
Have you attended any training course on sexuality education?		
Yes	41.7	56.7
No	58.3	43.3

The majority of teachers in the urban district were aged 25-35 (57.9 %), while the majority of teachers in the rural district were aged 46-55 (36.7 %) and 25-35 (30 %). In both districts, the majority of respondents were female (63.9% and 55.2 % of the respondents in the urban and rural districts were female respectively).

A majority of the responding teachers (42.1%) in the urban district reported belonging to Catholics, while the majority of those in the rural district (50%) reported belonging to Protestants. In both districts, more than 90 percent of the responding teachers reported attending religious services either *every day* or *at least once a week* and that religion was *very important* in their life.

The majority of teachers in the urban district (92.1%) and rural district (73.3 %) held a certificate in education (Grade A) as their teaching qualification. The majority of teachers in the urban district (42.1%t) had teaching experience of less than 5 years, while the majority of teachers in the rural district (53.3%) had teaching experience of more than 15 years. About 57 percent of teachers in the rural district indicated having attended some training programmes in teaching sexuality education, compared to 42 percent of teachers in the urban district.

### 2.2 The Instrument and Procedure

Teachers completed a questionnaire entitled *Teachers’ views and attitudes towards school-based sexuality education in Tanzania*. The questionnaire was initially prepared in English; it was then translated into Kiswahili, which is the working language for the majority of participants in the study sites. The Kiswahili version was translated back into English to ensure that the original content in the questionnaire was preserved.

The questions used in the questionnaire were adapted from the questionnaire Weaver et al. (2002) used in a similar study conducted in Canada. Although most of the questions adapted were developed in the context of Canada, they were found to be applicable in measuring the views and attitudes of teachers in Tanzania; the items that were included in the questionnaire had a good reliability, with an average Cronbach’s alpha of .93.

The questionnaire comprised three major sections. In section one, respondents were provided with two questions. In the first question, on a five response option ranging from *strongly disagree* (1) to *strongly agree* (5), teachers were asked to indicate to what extent they agreed that *sexuality education should be provided in schools in Tanzania*. In the second question, teachers were asked to indicate the school^1^ level at which they thought sexuality education should be introduced in schools; they were given five response options: *Class 4, Class 5-7, Form 1-2, Form 3-4* and *Form 5-6*.

In the third section of the questionnaire, teachers were provided with a list of 44 sexuality education topics and, on a five response option ranging from *Not at all important (1)* to V*ery important (5)*, were asked to indicate the level of importance they attached to each. In the fourth section, teachers were requested to provide some personal information, including age, educational level, religion, sex and marital status.

Data were entered and analysed using SPSS statistical package Version 16 using a guide provided by Pallant (2005). The percentages of respondents in favour of various aspects of school-based sexuality education were computed and were used to assess the extent to which teachers supported the provision of sexuality education in schools. Independent samples t-test was performed to explore the variation in the views and attitudes towards school-based sexuality education between teachers in the urban (Kinondoni) and rural (Sengerema) districts. Logistic regression analysis was performed to examine the effects of teachers’ demographic characteristics on their views and attitudes about school-based sexuality education. Multivariate statistical analysis (MANOVA) was conducted to explore the association between teachers’ demographics and their perceived importance of sexuality education topics.

## 3. Results

### 3.1 Teachers’ Attitudes towards Basic Issues Related to School-Based Sexuality Education

**Figure 1:**
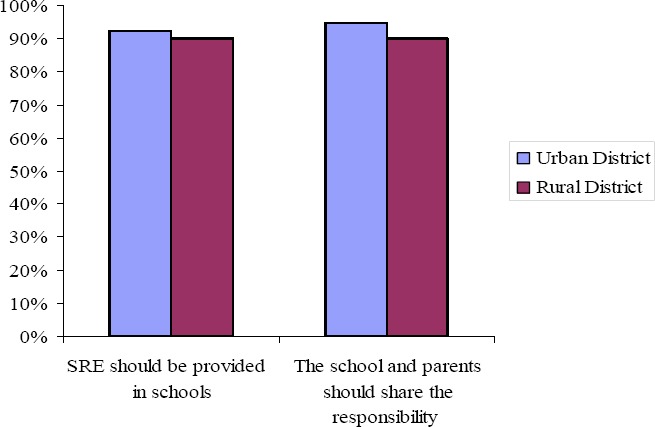
Percentage of teachers in the urban and rural districts agreeing with statements: sexuality education should be provided in schools and the school and teachers should share the responsibility

As can be seen in Figure 1, in both districts, an overwhelming majority of teachers supported the provision of sexuality education in schools. For example, 92.2 percent of teachers in the urban district (Kinondoni) agreed (42.1%) or strongly agreed (50.1%) with the statement that *sexuality education should be provided in schools*. In the rural district (Sengerema), 90 percent of teachers either agreed (30%) or strongly agreed (60%) with the statement.

**Figure 2:**
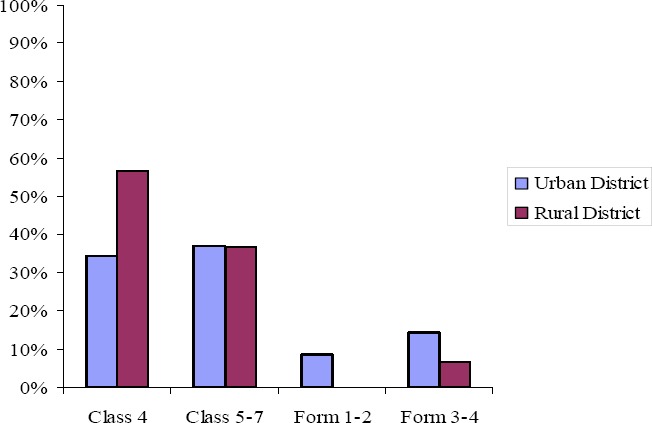
Percentage of teachers in the urban and rural districts indicating the level at which sexuality education should be introduced in schools

When asked to indicate the class level during which sexuality education should be introduced in schools, 71 percent of the teachers in the urban district and 94 percent of the teachers in the rural district indicated that sexuality education should begin at primary school level between Class 4 and 7 (see Figure 2). The paired- samples t- test revealed a statistically significant difference in teachers’ preference of the school level to introduce sexuality education between the primary school level (*M*=4.14, *SD*=1.12) and the secondary school level (*M*=2.39, *SD*=1.46): *t*(58)=6.224, *p*< .0005. The eta squared statistic (.40) indicated a large effect size. However, a one-way between groups MANOVA revealed no statistically significant difference in the preference of the school level to introduce sexuality education in schools between teachers in the urban and rural districts: *F*(2, 56)=2.053, *p* = .138; Wilks’ Lambda= .932.

### 3.2 Effects of Teachers’ Demographic Characteristics on their Attitudes towards School-Based Sexuality Education

A logistic regression analysis was performed with *sexuality education* *should be provided in schools* as Dependent Variable (DV) and *sex, teaching subject, experience of training in sexuality education, location* and *religion* as Independent Variables (IVs). A total of 198 cases were analysed. The examination of the Omnibus Tests of Model Coefficients showed that the full model did not statistically significantly predict teachers’ positive attitudes about the statement, *sexuality education* *should be provided in* *schools* (omnibus chi-square=5.938, df=6, *p*= .430). The model accounted for between 28.3% (Cox & Snell R Square= .096) and 21.7% (Nagelkerke R Square= .217) of the variance. However, the Hosmer and Lemeshow test showed that the model was accurate in predicting the teachers’ attitudes towards the provision of sexuality education in schools (Hosmer and Lemeshow test Chi-square=7.884, df=8, *p*= .445), with the model successfully predicting 91.5% of the respondents agreeing with the statement that *sexuality education should be provided in schools*.

Table 2 presents coefficients and the Wald statistic and associated degrees of freedom and probability values for each of the predictor variables. The table shows that none of the teachers’ demographic characteristics reliably predicted the teachers’ attitudes towards school-based sexuality education.

**Table 2:** Variables entered in the logistic regression equation with the resultant coefficients

Predictor variables		B	S.E.	Wald	df	Sig.	Exp(B)	95.0% C.I.for EXP(B)	
		Lower	Upper	Lower	Upper	Lower	Upper	Lower	Upper
Step 1(a)	sex(1)	1.675	1.330	1.584	1	.208	5.336	.393	72.403
	Teaching subject(1)	1.160	1.175	.975	1	.323	3.191	.319	31.919
	Training in Sexuality Education(1)	-2.504	1.428	3.075	1	.079	.082	.005	1.343
	Location (1)	-.024	1.091	.000	1	.982	.976	.115	8.281
	Age(1)	-1.054	1.130	.870	1	.351	.349	.038	3.191
	Religion (1)	-1.093	1.431	.584	1	.445	.335	.020	5.537
	Constant	3.779	1.574	5.767	1	.016	43.780		

a: Variable(s) entered on step 1: sex, Teaching subject, Training in sexuality education, Location, Age, Religion

### 3.3 Teachers’ Views on the Importance of Topics to Be Included In the School-Based Sexuality Education

Teachers were provided with a list of 44 sexuality education topics that are commonly included in a sexuality education curriculum, and were asked to rate the importance of each topic on a five-point scale ranging from *Not important at all* (1) to *Very important* (1).

As can be seen from Table 2, teachers rated the majority of topics as *very important* or *important* (median score of 5 and 4 respectively). For example, teachers in the urban district rated 32 topics (72.7 percent of all topics) as *very important* (18 topics) or *important* (14 topics). Teachers in the rural district rated 33 topics (75 percent of all topics) as *very important* (19 topics) or *important* (14 topics).

Teachers in the urban district rated nine topics as *somehow important* (median score =3), whereas teachers in the rural district rated six topics as such. Only two topics were rated by teachers in both districts as *not important at all*, these were *homosexuality* and *pornography*. *Sex as part of a loving relationship* was rated by teachers in both districts as *not important* (median score=2*)*. Additionally, teachers in the rural district rated five other topics as *not important* (median score=2); namely, *masturbation, appropriate/inappropriate touching, sexuality as a positive aspect of self, common myths about sexuality* and *sexual feelings and expression*.

**Figure 3:**
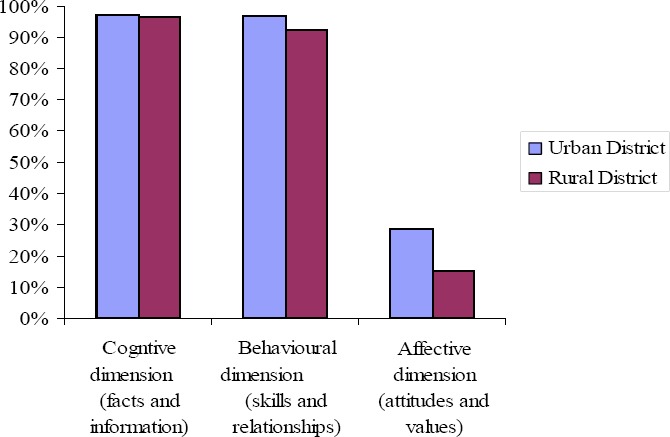
Percentage of teachers in the urban and rural districts indicating the importance of sexuality education dimensions

When the topics were classified into the three dimensions of sexuality education—cognitive, affective and behavioural dimensions—using a factor analysis (see Table 3 for items belonging to the three dimensions), it emerged that the affective dimension (encompassing topics on attitudes and values) was the least preferred by the majority of teachers in both districts. Whereas more than 90 percent of teachers in both districts rated cognitive (encompassing topics on facts and information) and behavioural (encompassing topics on skills and interpersonal relationships) dimensions as *very important* or *important*, less than 30 percent of teachers rated the affective dimension as such (see Figure 3).

**Table 3:** Components of SRE dimensions as extracted from rotated matrix of factor analysis with corresponding mean values in brackets

Cognitive dimension (facts and information)	Affective dimension (attitudes and values)	Behavioural dimension (relationships and interpersonal skills)
1. Menstruation (4.4)	1. Communicating about sex (3.0)	1. Sexual behaviours other than intercourse (3.0)
2. Puberty (4.5)	2. Attraction, love and intimacy (3.1)	2. Masturbation as an alternative to sexual intercourse (2.7)
3. Body image (4.1)	3. Sex as part of a loving relationship (2.1)	3. Appropriate/inappropriate touching (3.2)
4. Pregnancy (3.9)	4. Sexual behaviours (3.5)	4. Common myths concerning sexuality (3.7)
5. Correct names of genitalia (3.5)	5. Homosexuality (2.5)	5. Abstinence as an alternative to sexual intercourse (3.7)
6. Wet dreams (3.4)	6. Pornography (1.9)	6. Reduction of fears and myths about sexuality matters (3.8)
	7. Being comfortable with the other sex (3.6)	7. Decision making (4.4)
	8. Masturbation (3.4)	

(Note: Response options: 1=Not at all important, 2=Not important, 3=Somehow important, 4=Important and 5=Very important)

### 3.4 Variation in the Attitudes towards Sexuality Education between Rural and Urban Teachers

An independent samples t-test was performed to investigate the variation in attitudes towards school- based sexuality education between rural and urban teachers, with *location* as independent variable and *sexuality education should be provided in schools* as dependent variable. There was no statistically significant difference in scores measuring the attitudes towards school- based sexuality education between rural teachers (*M*=4.43, *SD*= .86) and urban teachers (*M*=4.32, *SD*= .93); *t*(66)=- .534, *p*= .59, with a very small magnitude of the difference in the means (eta squared= .004).

A one-way between groups ANOVA was performed to investigate variation in the perceived importance of the three mostly commonly identified controversial topics in school-based sexuality education: *homosexuality, masturbation* and *condom use*. These are the topics that received the lowest rating, with clear percentage variation between urban and rural teachers. Preliminary assumption testing using homogeneity of variance-covariance matrices revealed no serious violation of this assumption. There was no statistically significant difference between urban and rural based responding teachers on the combined dependent variables: *F*(3, 57)= .90, *p*= .442; Wilkis’ Lambda= .95; partial eta squared= .05. Thus, despite the actual percentage variation, there was no statistically significant difference in attitudes towards these topics between urban and rural based teachers.

### 3.5 Teachers’ Preferred Class Levels to Introduce Sexuality Education Topics

Teachers were asked to indicate the class levels to introduce each of the 44 topics in the school-based sexuality education curriculum. There were five response options: Class 4 (age 10), Class 5-7 (age 11-13), Form 1-2 (age 14-15), Form 3-4 (age 16-17) and Form 5-6 (age 18-20). In accordance with the education system in Tanzania, primary and secondary education levels roughly correspond to ages 7-13 and 14-17 respectively. In practice, however, a significant number of children complete primary education much later than age 13.

Tables 4 summarises the teachers’ preference of school levels for introducing 12 sexuality education topics that have been considered as constituting the minimum package for a school- based sexuality education programme (Byers et al., 2003; Lenderyou, 1993). The table shows that the majority of teachers wanted most of the topics to be introduced at some point during the children’s primary education (ages 10-13). For example, in both districts, the overwhelming majority of the teachers wanted all but one topic (*homosexuality*) to be introduced during primary school between Classes 4 and 7 (ages 10 and 13). In both districts, the majority of teachers wanted *STDs/HIV/AIDS* to be introduced at a much earlier level than other topics; 55.3 percent of the teachers in the urban district and 43.3 percent of the teachers in the rural district indicated that this topic should be introduced at Class 4 (age 10).

**Table 4:** Percentage of teachers indicating the class levels for introducing sexuality education topics by district

	Percentage of teachers indicating each class level									

	% Urban District (Kinondoni) N=82-102					% Rural District (Sengerema) N=80-96				

Topics	Class 4	Class 5-7	Form 1-2	Form 3-4	Form 5-6	Class 4	Class 5-7	Form 1-2	Form 3-4	Form 5-6
Names of genitalia	20.6	50.0	14.7	8.8	5.9	10.7	71.4	14.3	0.0	3.6
Personal safety	43.2	43.2	2.7	5.4	5.4	26.7	66.7	6.7	0.0	0.0
Puberty	10.8	75.7	2.7	5.4	5.4	16.7	73.3	3.3	0.0	6.7
Reproduction and birth	8.8	58.8	14.7	11.8	5.9	17.2	62.1	17.2	3.4	0.0
Abstinence	7.7	50.0	26.9	11.5	3.8	0.0	43.5	34.8	21.7	0.0
Sexual pleasure	0.0	73.3	10.0	10.0	6.7	7.7	61.5	15.4	15.4	0.0
Decision making	0.0	56.0	16.0	24.0	4.0	3.8	69.2	19.2	7.7	0.0
Condom use	3.6	46.4	14.3	28.6	7.1	7.7	53.8	3.8	34.6	0.0
STDs and HIV/AIDS	55.3	28.9	7.9	0.0	0.0	43.3	40.0	10.0	6.7	0.0
Sexual coercion	29.7	56.8	2.7	2.7	8.1	30.0	70.0	0.0	0.0	0.0
Masturbation	10.3	51.7	10.3	20.7	6.9	0.0	54.2	25.0	20.8	0.0
Homosexuality	15.6	21.9	18.8	18.8	9.4	25.9	14.8	11.1	29.6	0.0

### 3.6 Teachers’ Confidence in Teaching Various Sexuality Education Topics

Teachers were asked to indicate the extent to which they would find it easy or difficulty to teach sexuality education topics in schools on a five response scale ranging from “*very difficult (1)* to *very easy (5)*”. Table 5 summarises the teachers’ responses regarding their views about the comfort in teaching each of the 12 basic sexuality education topics. As shown in the table, teachers were divided regarding their level of comfort in teaching sexuality education topics; in both districts, teachers expressed easiness in teaching some topics and difficulty in many others.

**Table 5:** Percentage of teachers indicating the extent to which they would find it easy or difficult to teach various sexuality education topics

	% Teachers Urban District (Kinondoni) N=81-102					% Teachers Rural District (Sengerema) N=80-96				

	Very difficult	Difficult	Neutral	Easy	Very easy	Very difficult	Difficult	Neutral	Easy	Very easy
Correct names of genitalia	25.0	11.1	30.6	22.2	11.1	13.8	17.2	10.3	41.4	17.2
Personal safety	0.0	2.8	8.3	38.9	50.0	0.0	3.4		58.6	37.9
Puberty	2.8	5.6	8.3	69.4	13.9	0.0	3.6	21.4	39.3	35.7
Reproduction and birth	11.8	11.8	26.5	26.5	23.5	0.0	10.0	0.0	40.0	50.0
Abstinence	13.9	11.1	16.7	33.3	25.0	19.2	7.7	26.9	26.9	19.2
Sexual pleasure and enjoyment	22.9	5.7	28.6	25.7	17.1	21.4	7.1	17.9	17.9	35.7
Sexual decision making	2.8	0.0	13.9	41.7	41.7	3.4	6.9	0.0	34.5	55.2
Condom use	36.1	16.7	19.4	19.4	8.3	24.1	13.8	10.3	20.7	31.0
STDs/HIV/AIDS	2.8	2.8	19.4	27.8	47.2	3.4	0.0	0.0	34.5	62.1
Sexual coercion and assault	0.0	0.0	16.7	38.9	44.4	3.6	7.1	7.1	32.1	50.0
Masturbation	25.7	17.1	25.7	22.9	8.6	32.1	7.1	25.0	21.4	14.3
Homosexuality	36.1	13.9	30.6	13.9	5.6	25.9	22.2	22.2	18.5	11.1

Only four topics were seen by teachers in the urban district (Kinondoni) as easy to teach, with more than 80 percent of the teachers indicating that these topics were either *very easy* or *easy* to teach. The topics and the percentage (in brackets) of teachers indicating that they would find them very easy and easy to teach are *personal safety* (88.9%), *puberty* (83.3%), *sexual decision making* (83.3%) and *sexual coercion and assault* (83.3%). Teachers in the rural district (Sengerema) indicated five topics as *very easy* or *easy* to teach; namely, *puberty* (96.6%), *reproduction and birth* (90.0%), *sexual decision making* (89.75), *STDs and HIV/AIDS* (96.6%) and *sexual coercion and assault* (82.1%).

Teachers in the urban district expressed difficult in teaching *correct names of genitalia, sexual pleasure and enjoyment, condom use, masturbation* and *homosexuality*, with less than 40% of them indicating that they would find it *very easy* or *easy* teaching these topics. Interestingly, teachers in the rural district indicated only two topics (*masturbation* and *homosexuality*) as being very difficult or difficult in teaching, with less than 40 percent of them indicating that they would find it very difficult or difficult in teaching these topics.

As would be expected, *homosexuality* was seen by teachers in both districts as the most difficult topic to teach, with 50 percent of the teachers in the urban district (Kinondoni) indicating that they would find it very difficult (36.1%) or difficult (13.9%) teaching this topic. In the rural district (Sengerema), 48.1 percent of the teachers indicated that they would find it very difficult (25.9%) or difficult (22.2%) teaching *homosexuality*.

Again, the one-way between groups MANOVA revealed no statistically significant difference between urban and rural based responding teachers in their comfort about teaching the most three controversial sexuality education topics (*homosexuality*, *masturbation* and *condom use*): *F* (3, 55)= 1.473, *p*= .232; Wilkis’ Lambda= .926.

## 4. Discussion

The results of this study show that an overwhelming majority of the responding teachers in both rural and urban districts supported the provision of comprehensive school-based sexuality education. Furthermore, the results show that the majority of the teachers who took part in the study wanted sexuality education to begin early during primary education (ages 10-13) rather than during secondary education (ages 14 and above).

Again, teachers supported a wide range of sexuality education topics to be included in the school curriculum. This implies that teachers view school-based sexuality education not only as an important strategy for protecting young people from HIV/AIDS and other sexual health problems (diseases prevention model), but as an important strategy for promoting healthy adolescent sexual development.

The results of this study have also shown that though teachers may be committed to teaching sexuality education in schools, they are currently incapacitated to do so by the low status of sexuality education in the school curriculum. Nevertheless, this may not be the only factor behind the poor teaching of sexuality education in schools as teachers also showed considerable anxieties in teaching several topics. For example, when the teachers were presented with the same topics they had rated as important and asked to indicate their comfort about teaching them, a majority of the teachers expressed uneasiness in teaching many of the topics. The majority of teachers in the urban district, for example, expressed comfort in teaching only four topics; namely, *personal safety, puberty, decision making* and *sexual coercion and assault*. Teachers in the rural district expressed comfort in teaching only five topics: *puberty, reproduction and birth, decision making, STDs and HIV/AIDS* and *sexual coercion and assault*.

These results show that though teachers may support the teaching of sexuality education and the inclusion of a number of topics in the school curriculum, they may not be comfortable and capable of teaching all the key sexuality education topics. This was particularly the case with regard to *homosexuality* and other controversial topics. This implies that adequate preparation in a way of training is required for teachers if they are to handle sexuality education in the classroom situation effectively. Therefore declaration of positive attitudes towards school-based sexuality education as well as change in policy alone may not be enough; these need to go hand in hand with providing teachers with the knowledge, skills and confidence to teach the various sexuality education topics.

The results of this study are in line with the findings of previous studies, both in developing and developed countries. For example, a study in Nigeria (Iyoke, Onah, & Onwasigwe, 2006) concluded that teachers’ attitude was not an impediment to implementing sexuality education. Rather, it is the level of comfort and skills in handling the various sexuality education topics in the classroom context that may constrain teachers’ effective delivery of the programme. Furthermore, a study in America (Bowden et al., 2003) came up with similar conclusions; the majority of teachers overwhelmingly supported the teaching of comprehensive sexuality education in schools, but they needed training to build their confidence and skills needed to handle sexuality education topics in classes.

The lack of variation in attitudes towards the provision of sexuality education in schools between urban and rural based teachers is revealing. This may indicate that the level of education about sexual health between urban and rural localities is now becoming uniform. Additionally, the apparent lack of variation may be attributed to increased access to electronic and print media in rural areas, where there is almost an equal access to these media between urban and rural residents. Further, in many African countries, the initial stages of HIV and AIDS campaigns were more intense in rural areas than in urban areas.

## 5. Conclusion

The results of this have clearly shown that the overwhelming majority of teachers, in both rural and urban localities, support the provision of sexuality education in schools, as well as the inclusion of a wide range of sexuality education topics in the school curriculum. Nevertheless, the majority of teachers are not comfortable in teaching most of the topics to schoolchildren. This implies that there various strategies are needed in ensuring that the provision of sexuality education in schools in Tanzania is successful, including using other professionals such as physicians and nurses to assist teachers in facilitating some of the sexuality education topics, as well as retraining teachers in the area of sexual and reproductive health.

## Footnotes

1 According to the Tanzanian education system, primary education consists of seven years from Standard (also called Class) 1 to 7 (ages 7-13). Secondary education consists of ordinary and advanced levels, with the ordinary secondary school level consisting of four years from Form 1 to 4 (ages 14-17) and the advanced secondary level consisting of two years- Form 5 and 6 (ages 18-19)
